# Ultrahigh frequency transcutaneous electrical nerve stimulation for neuropathic pain alleviation and neuromodulation

**DOI:** 10.1016/j.neurot.2024.e00336

**Published:** 2024-02-16

**Authors:** Szu-Han Chen, Yu-Wen Lin, Wan-Ling Tseng, Wei-Tso Lin, Sheng-Che Lin, Yuan-Yu Hsueh

**Affiliations:** a Division of Plastic and Reconstructive Surgery, Department of Surgery, National Cheng Kung University Hospital, College of Medicine, National Cheng Kung University, Tainan, Taiwan; b Center of Cell Therapy, National Cheng Kung University Hospital, College of Medicine, National Cheng Kung University, Tainan, Taiwan; c International Research Center for Wound Repair and Regeneration, National Cheng Kung University, Tainan, Taiwan; d Institute of Clinical Medicine, College of Medicine, National Cheng Kung University, Tainan, Taiwan; e Division of Plastic and Reconstructive Surgery, Department of Surgery, Tainan Hospital, Ministry of Health and Welfare, Tainan 700, Taiwan; f Gimer Medical Co., Ltd, New Taipei City, Taiwan; g Division of Plastic Surgery, Department of Surgery, An-Nan Hospital, China Medical University, Tainan, Taiwan

**Keywords:** Neuropathic pain, Compression neuropathy, Transcutaneous electrical nerve stimulation, Ultrahigh frequency, Neuroinflammation

## Abstract

A challenging complication in patients with peripheral compressive neuropathy is neuropathic pain. Excessive neuroinflammation at the injury site worsens neuropathic pain and impairs function. Currently, non-invasive modulation techniques like transcutaneous electrical nerve stimulation (TENS) have shown therapeutic promise with positive results. However, the underlying regulatory molecular mechanism for pain relief remains complex and unexplored. This study aimed to validate the therapeutic effect of ultrahigh frequency (UHF)-TENS in chronic constriction injury of the rat sciatic nerve. Alleviation of mechanical allodynia was achieved through the application of UHF-TENS, lasting for 3 days after one session of therapy and 4 days after two sessions, without causing additional damage to the myelinated axon structure. The entire tissue collection schedule was divided into four time points: nerve exposure surgery, 7 days after nerve ligation, and 1 and 5 days after one session of UHF therapy. Significant reductions in pain-related neuropeptides, MEK, c-Myc, c-FOS, COX2, and substance P, were observed in the injured DRG neurons after UHF therapy. RNA sequencing of differential gene expression in sensory neurons revealed significant downregulation in Cables, Pik3r1, Vps4b, Tlr7, and Ezh2 after UHF therapy, while upregulation was observed in Nfkbie and Cln3. UHF-TENS effectively and safely relieved neuropathic pain without causing further nerve damage. The decreased production of pain-related neuropeptides within the DRG provided the therapeutic benefit. Possible molecular mechanisms behind UHF-TENS may result from the modulation of the NF-κB complex, toll-like receptor-7, and phosphoinositide 3-kinase/Akt signaling pathways. These results suggest the neuromodulatory effects of UHF-TENS in rat sciatic nerve chronic constriction injury, including alleviation of neuropathic pain, amelioration of pain-related neuropeptides, and regulation of neuroinflammatory gene expression. In combination with the regulation of related neuroinflammatory genes, UHF-TENS could become a new modality for enhancing the treatment of neuropathic pain in the future.

## Graphical abstract

**Figure:**
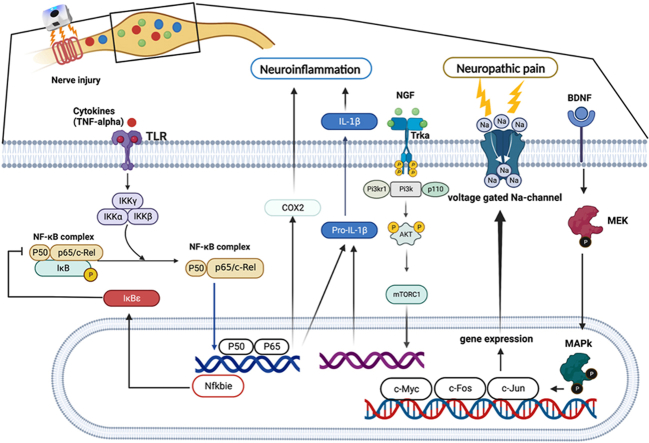

## Introduction

Neuropathic pain, a condition caused by acute or chronic injury to the somatosensory nervous system, results from several pathological sequelae, which are typically manifested based on anatomical localization or etiology [1]. Neuropathic pain is primarily associated with diabetes mellitus, viral infections, autoimmune disorders, chemotherapy, and trauma [2]. The signs and symptoms of neuropathic pain include allodynia, hyperalgesia, and paresthesia. Patients suffering from neuropathic pain often experience a decreased quality of life, negatively affecting their psychosocial state [3]. The prevalence of neuropathic pain is approximately 7%–8% within the general population, accounting for 20%–25% of individuals with chronic pain [3,4]. Current treatments for neuropathic pain can be categorized into pharmacological and non-pharmacological treatments, including interventional, physical, and psychological therapies [1,5]. Neuropathic pain management focuses on symptom alleviation; however, evidence suggests that these pain management drugs have poor efficacy and often do not provide sufficient pain relief at recommended doses, which can also lead to severe adverse effects [6]. Conversely, non-pharmacological interventions for neuropathic pain remain controversial, with weak recommendations based on previous studies [[7], [8], [9]].

The complexity of the underlying mechanisms of neuropathic pain leads to unsatisfactory results with current treatments. Available research evidence indicates that excessive neuroinflammation contributes to neuropathic pain [10]. After nerve injury, pain mediators such as cyclooxygenase 2 (COX2) and prostaglandin E2 (PGE2) are persistently produced in invading macrophages and Schwann cells in injured axons. PGE2 induces the synthesis of pain peptides like Substance P (SP), Calcitonin gene-related peptide, interleukin (IL)-6, and the neurotrophin factor brain-derived neurotrophic factor (BDNF) in injured dorsal root ganglion (DRG) [11]. During an inflammatory reaction following nerve injury, BDNF is produced, and ligand receptors are activated by G-proteins Ras, Raf, and MAP kinase (MAPK) within the traumatized nerve tissue. These activated receptor complexes anterogradely induce the phosphorylation of MAPK in the DRG neurons. After phosphorylation, a component of activated MAPK relocates to the nucleus, where other phosphorylation events activate the following transcription factors, including c-Myc, Elk-1, c-Fos, and c-Jun. These processes eventually contribute to dynamic nuclear remodeling, prolonging potentiation and decreasing receptor activation threshold, leading to clinical neuropathic pain [12].

To provide significant benefits for patients with stroke or spinal cord injury, electrical stimulation applications are among the most promising methods for neuromodulation. Electrical stimulation can exercise paralyzed muscles, reverse atrophy, improve cardiovascular function, and reduce progression of osteoporosis. Other potential therapeutic uses being investigated include reduction of spasticity, prevention of deep vein thrombosis, and improvement of respiratory, bladder, bowel, and sexual functions [13,14]. Transcutaneous electrical nerve stimulation (TENS) is a nonpharmacological intervention in attenuating neuropathic pain in patients [15]. It has value by virtue of its accessibility and safety, but the absence of high-quality evidence of the analgesic benefit of TENS devices for neuropathic pain and a high degree of heterogeneity in applied waveforms (e.g., intensity, frequency, or proprietary patterns) were noted [16]. In rodent model of neuropathic pain, TENS may be a promising therapy, but caution is advised due to the high risk of bias in the included studies, such as omitted housing method, omitted blinded fashion, and so on. In addition, most included articles used 100Hz frequency of electrical stimulation. The use of electrical stimulation with ultrahigh frequency (∼500 ​KHz) is relatively rare, and the efficacy and possible mechanism behind it were still uncertain [17]. In previous study, pulse ultrahigh frequency (∼500 ​KHz)- spinal cord stimulation (UHF-SCS) inhibited neuropathic pain–related behavior distinct from low frequency-SCS [18]. UHF-SCS could potentially impact intracellular signaling and synaptic plasticity through various mechanisms. These include the potential for increased expression of the Fos proto-oncogene AP-1 transcription factor subunit (c-Fos) [19], decreased levels of presynaptic excitatory neurotransmitters [20] and glia-derived kinases in the spinal cord [18], activation of the descending pain-inhibitory pathways [21], regulation of pain pathway gene expression [22], and activation of the endogenous opioidergic system [23]. However, the efficacy and molecular mechanism of UHF-TENS for peripheral neuropathic pain remain unclear and unexplored. In this study, we aim to validate the therapeutic effect of UHF application through a newly designed TENS apparatus and explore the underlying molecular mechanisms accordingly.

## Materials and Methods

### Animals and surgical procedures

The Laboratory Animal Center and Institutional Animal Care and Use Committee No. 111224 at National Cheng Kung University (Tainan, Taiwan) approved the animal protocols and surgical procedures such as animal housing and care. Adult Sprague–Dawley male rats, weighing 250–300 ​g, were used in this study. Anesthesia was induced via inhalation of 3% isoflurane in air (USP, Sigma-Aldrich, St. Louis, MO), which was followed by the toe pinch test to assess for reactivity and was maintained at 1.5%–2% isoflurane in air. For the biosafety test, we used a total of 13 rats without employing CCI but underwent nerve exposure surgery. Among them, 6 were divided into groups of three, with and without treatment, for von Frey test. The remaining 7 rats were divided into groups of four without treatment and three with treatment, for immunofluorescent staining (IF) of nerve. For the definite treatment, a total of 44 rats, which comprised 12 rats for von Frey test, 32 rats for IF and RNA sequencing, were used. Among them, 8 rats for sham group underwent only nerve exposure surgery without ligation, and the remaining 36 rats underwent chronic constriction injury (CCI) model on the rat sciatic nerve by computer monitoring of controllable forces (6 ​g string tension) (Supplementary Table 1). A week later, the compressive neuropathy of the modified CCI model had significant and persistent mechanical allodynia and increased neuroinflammation, following the protocol established in our previous studies [24,25]. The left sciatic nerve was dissected from the circumambient tissues and exposed at the middle level of the thigh. The area proximal to the trifurcation of the sciatic nerve was freed from the adhering tissue. Four loosely constricting ligatures with 5-0 Nylon (Ethicon US, Bridgewater, NJ) were tied 1 ​cm above the sciatic trifurcation with approximately 1 ​mm between each ligature. After surgery, the thigh muscles and skin were sutured, and the rat was placed back into the cage without anesthesia for recovery.

### UHF-TENS for the injured sciatic nerve

StimOn™ Pain Relief System (GM2439, Gimer Medical Co., Ltd, New Taipei City, Taiwan; FDA 510(k) No. K213802) is a wearable TENS device with innovative UHF technology aimed at alleviating symptomatic chronic pain that does not produce muscle twitch during stimulation. The UHF intervention protocol was mainly based on previous studies, which revealed that UHF (500-KHz sine wave, 2-Hz frequency with 25-ms pulse width) could attenuate mechanical allodynia by selectively and persistently modulating C-fiber-mediated spinal nociceptive hypersensitivity [26,27].

**Fig. 1:**
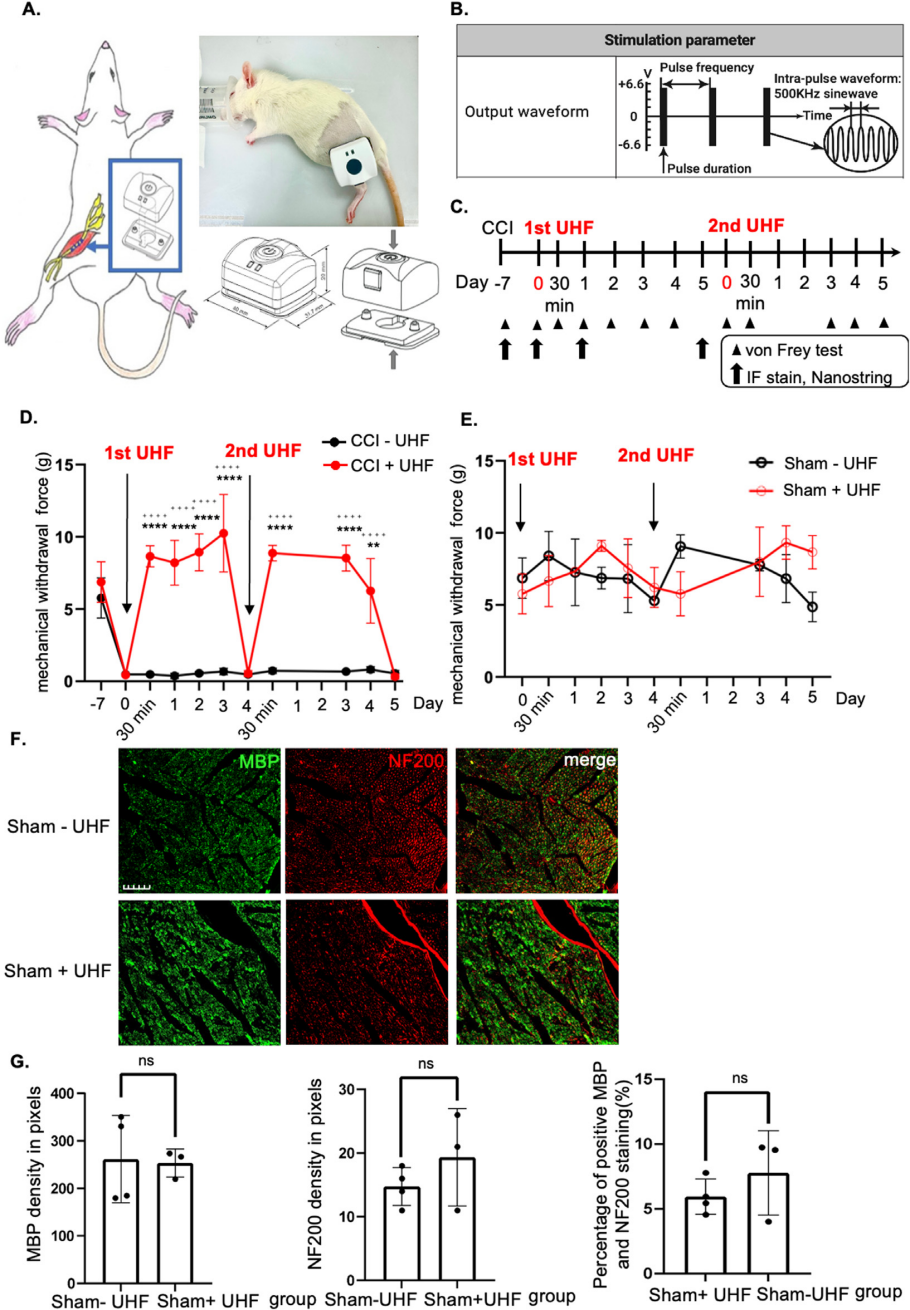
Functional behavior outcome by ultrahigh frequency transcutaneous electrical stimulation therapy. (A) Animal model of chronic constriction injury over the ipsilateral sciatic nerve. The device of UHF-TENS was applied on the skin of the treated side sciatic nerve under stimulation protocol. (B) Stimulation parameter and output waveform in UHF (C) Timeline and study design of UHF-TENS treatment in this study. (Arrowhead: time point for von Frey test; Arrow: time point for tissue collection for IF and nanostring) (D, E) The mechanical withdrawal force (g) indicated the forces that induced paw withdrawal by the Von Frey test for injured or uninjured nerves with or without UHF treatment. (n ​= ​6 per group in injured group; n ​= ​3 per group in uninjured group) Data were presented with mean ​± ​standard deviation, ∗∗∗∗ indicated statistical significance in UHF group as compared to the no UHF group; p ​< ​0.0001; ^++++^ indicated statistical significance at different time point as compared to day 0 in UHF group). (F, G) Immunofluorescent staining of uninjured sciatic nerve group. Mature axon staining with NF200 revealed no significant difference between the uninjured group with and without electrical stimulation therapy. Similar pattern was also observed in Schwann cell staining with MBP alone and colocalization image. NF200/MBP colocalization revealed that the amount of myelinated axon after UHF in the uninjured nerve group was no significant difference in the uninjured nerve group without UHF stimulation. (n ​= ​3 in UHF group and n ​= ​4 in control group; Scale bar: 50 ​μm; ns indicated no significant difference).

The TENS device was attached to the electrode pad through magnetic attraction. Then, we placed the device above and perpendicular to the direction of the underlying sciatic nerve, ensuring that the electrode pad was firmly attached to the incision area (StimOn™ Pain Relief System [GM2439], Gimer Medical Co., Ltd., Taiwan). A 500-KHz stimulus was delivered. The symmetric two-phase sine wave has a 2-Hz frequency with a 25-ms pulse width (Fig. 1A). The treatment session lasted for 15 ​min. The output peak current was 13.2 ​mA and the output peak voltage was 6.6 ​V at 500 ​Ω. When the nerve stimulation was conducted, the rats were under anesthesia with inhaled isoflurane. Thus, the training sessions to accommodate the animals were not necessary.

### Sensory assessment

To evaluate mechanical allodynia, the von Frey test was used to measure the threshold force required to elicit paw withdrawal. The rats were placed on an elevated wire mesh platform for 5 ​min until they were acclimatized in the testing environment. A series of 20 Semmes–Weinstein monofilaments (SWM; North Coast Medical, Inc., Morgan Hill, CA), (1.65/0.08 ​g, 2.36/0.02 ​g, 2.44/0.04 ​g, 2.83/0.07 ​g, 3.22/0.16 ​g, 3.61/0.4 ​g, 3.84/0.6 ​g, 4.08/1 ​g, 4.17/1.4 ​g, 4.31/2 ​g, 4.56/4 ​g, 4.71/6 ​g, 4.93/8 ​g, 5.07/10 ​g, and 5.18/15 ​g, and starting from the 4 ​g) were inserted through the mesh and the underside of a hind paw was poked, using an up-down method [28]. A monofilament was applied until it buckled, delivering a constant predetermined force for 2–5 ​s. The animals responded by flicking their paw away from the hair when the threshold was reached. The withdrawal forces of the hindlimb were measured by the researcher who was blinded to the experimental conditions; thereafter, analysis was conducted to quantify the allodynia. The threshold value was the average of the two measurements, separated by at least 10–15 ​min.

A total of 18 rats were used In our experiments. Among them, 12 and 6 rats were used for the uninjured and injured groups, respectively. The uninjured group received only nerve exposure surgery, while nerve ligation was conducted in the injured group. For both the uninjured and injured groups, behavioral tests were conducted on several time points (day −7, before nerve exposure surgery or nerve ligation; day 0, before first stimulation; 30 ​min, D1, D2, D3, and D4 after 1st stimulation; 30 ​min, D3, D4, and D5 after 2nd stimulation; compared with no UHF stimulation) (Fig. 1C).

### Immunofluorescent staining (IF)

The animals were sacrificed under deep anesthesia with isoflurane and transcardially perfused in phosphate-buffered saline (PBS). The middle segment of the constricted sciatic nerve was obtained to perform myelin basic protein (MBP)/NF200 staining for the sham group with stimulation, compared with the no stimulation group (n ​= ​3 in sham ​+ ​UHF group and n ​= ​4 in sham - UHF group) (Fig. 1F). In addition, ipsilateral L4–L5 dorsal root ganglion (DRG) of total 32 rats was obtained on D-7, D0, D1, and D5 for both histological analysis (n ​= ​4 per group) and RNA sequencing (n ​= ​4 per group) (Fig. 1C, Supplementary Table 1). The DRG obtained on D-7 was defined as the sham group which received nerve exposure surgery but no nerve ligation, D0 as the control group which underwent CCI for 7 days but did not receive electrical stimulation, D1 as the Day 1 group which underwent CCI and DRG extracted 1 day after treatment, and D5 as the Day 5 group which underwent CCI and DRG extracted 5 days after treatment.

The constrictive sciatic nerve and ipsilateral L4–L5 DRG were harvested and post-fixed in 10% paraformaldehyde for 2 ​h. The tissues were embedded and deep-frozen with an optimal cutting temperature (Tissue-Tek®, Sakura Finetek Inc, Torrance, CA, USA) until ready for use. Then, the embedded tissues of the sciatic nerve and DRG were sectioned in a cryostat (Leica CM1860) at a 10- and 16-μm thickness, respectively. The sections were washed in PBS three times (10 ​min/wash), permeabilized using 0.5% Triton X-100 for 30 ​min, washed again in PBS twice, and blocked with 3% bovine serum albumin for 1 ​h. Next, one nerve section from each of the seven rats in total were incubated with mouse anti-neurofilament heavy polypeptide (NF200, N0142, 1:200; Sigma-Aldrich, St. Louis, MO, USA) and rabbit anti-MBP, GTX133108, 1:200; GeneTex, Irvine, California, USA) as primary antibodies. For L4–L5 DRG, a total of 82 sections were probed with the following antibodies: rabbit anti-c-FOS (GeneTex, GTX129846, 1:100), rabbit anti-brain-derived neurotrophic factor (BDNF) (Elabscience, E-AB-63470, 1:1000), rabbit anti-cyclooxygenase 2 (COX2) (Elabscience, E-AB 62884, 1:200), rabbit anti-c-Myc (GeneTex, GTX17356, 1:100), guinea pig anti-substance P (SP) (GeneTex, GTX10353, 1:200), and rabbit anti-MEK1 (GeneTex, GTX134234, 1:100). After washing in PBS, the nerve and DRG sections were further incubated with secondary antibodies, goat anti-mouse IgG antibody (GeneTex, GTX213111-05, 1:200) for NF200, goat anti-guinea pig IgG antibody (GeneTex, GTX26965, 1:200) for SP, and goat anti-rabbit IgG antibody (GeneTex, GTX213110-04, 1:100) for BDNF, COX2, c-Myc, MEK, and c-FOS for 2 ​h at room temperature. After being washed, the DRG sections were incubated with diamidino-phenyl-indole (DAPI) for nucleus staining for 1 ​min. Then, all sections were washed three times in 0.01 ​M PBS and then cover-slipped with Mounting Medium with DAPI (Abcam, ab104139). These sections were further examined under a fluorescence microscope (BX61, Olympus, Tokyo, Japan) to analyze the protein expression level. Images were analyzed using ImageJ software (1.53s version, NIH, Bethesda, MD, USA). As for neuropeptide quantification, including BDNF, COX2, c-Myc, SP, c-FOS, and MEK, neurons that were stained after merging with DAPI and exhibited strong signals were defined as positive staining, while neurons with weak signals were defined as negative staining. Then, to calculate positive (indicated by solid arrowheads) and negative neurons (indicated by hollow arrowheads) based on different antibodies corresponding to a different color, ImageJ software was used. Quantification was performed by a researcher who was blinded to the group to determine the percentage of positive neurons compared to the total neurons within the DRG.

### Nanostring analysis for RNA sequencing

The method of RNA extraction and nanostring analysis followed our previous study with modification [24]. Total RNA was extracted from L4–L5 DRG of 16 rats (4 rats in each of group sham, control, day 1, and day 5) (Fig. 1C, Supplementary Table 1) using TRIzol (Sigma-Aldrich, St. Louis, MO, USA) as per the manufacturer's instructions. The resulting RNA was precipitated using 2-propanol, washed with 75% ethanol twice, and then dissolved in diethylpyrocarbonate-treated water to obtain a suitable volume. The total RNA concentration was determined by measuring the OD values of the samples at 260 ​nm.

The total extracted RNA from L4–L5 DRG was run on nCounter® Mouse Neuroinflammation v1.0 panel (NanoString Technologies, Seattle, WA) to simultaneously measure RNA transcript counts of 757 genes and 13 housekeeping genes. NanoString categorized these genes into 23 pathway annotations. To decide concentration and chemical purity (A260/230 and A260/280 ratios), RNA sample quality was confirmed by spectrophotometry (QuickDrop SpectraMax, Molecular Devices, San Jose, CA, USA) and with a bioanalyzer (model 2100, Agilent Technologies, Santa Clara, CA, USA). The mean value of A260/280 in the sham, control, day 1, and day 5 groups were 1.67, 1.80, 1.80, and 1.82, respectively. The determination of RNA quality to input RNA was based on DV300 calculation. The DV300 calculation determined the percentage of RNA fragments that are larger than 300 nucleotides in length with respect to all RNA fragments contained in the sample. The most reproducible and highest quality data were typically achieved with samples that have at least 50% of the RNA present greater than 300 nucleotides. Among four rats in each group, 3 rats were selected to be of better quality based on the DV300 value.

Sample gene transcript counts were normalized before downstream analysis by geNorm algorithm in NanoString's nSolver software version 4.0 to identify stable housekeeping genes and positive control for normalization. R language, which used RStudio Version February 1, 5033, was used to perform NanoString data analysis. Differential expression analysis was performed in each comparison using R with the “NanoStringDiff” package version 1.18.0, and statistically significant differentially expressed (DE) genes were set at a p-value <0.05.

NanoString's nSolver software was also used to calculate “Global Significance Scores (GSS)” for a group of samples by calculating the pattern of differential expression among all genes belonging to a particular neuroinflammatory pathway. The “Directed Global Significance Score” was compared to GSS as this might render either negative or positive values (for downregulated and upregulated pathways, respectively). A greater magnitude of directed GSS would be considered as a stronger pattern in the pathway level expression changes, and because these scores have been scaled to the same distribution (that of the t-statistic), they would be more robust to the comparisons between different pathways or experiments. A high score indicated that a large proportion of the genes in a pathway are exhibiting changes in expression across groups of samples.

Pathway scores were calculated into a small number of scores to summarize each sample's gene expression. Through the pathway scores, a simplified investigation could be further analyzed, rather than in higher-dimension lens of gene expression values. To calculate the pathway scores, the first principal component of each gene set's data was used, and the scores were set to ensure that an increase in score corresponds to an increase in expression (at least half of the genes in each pathway score had positive weights). In the pathway analysis, covariate plots to compare pathway scores to covariates were also used.

### Statistical analysis

Prism (ver. 9.5.1, GraphPad Software LLC., Boston, MA) was used for the experimental design and data analysis. To achieve significance, the sample size necessary t was estimated by utilizing Power and Precision statistical software (Englewood, NJ) with the following information: minimum significant effect to be detected, data variation, power (0.8), and type I error rate (0.05). Student's *t*-test was used to compare continuous data between two samples. To identify a statistically significant difference between groups with different managements, analysis of variance was performed for multiple sample comparisons, and post Hoc analysis was used. All experimental data are expressed as the mean ​± ​standard deviation (SD), and statistical significance (two-tailed) was set at a p-value of 0.05 or less.

## Results

### UHF-TENS temporarily alleviates neuropathic pain from CCI

To investigate the therapeutic effects of TENS for neuropathic pain in peripheral compressive neuropathy, a modified CCI model on rat sciatic nerve was utilized according to our previous researches [24,25]. Persistent mechanical allodynia was consistently induced at 1 week following unilateral nerve constriction injury (Fig. 1A). After confirming the induction of mechanical allodynia, two sessions of UHF-TENS were administered (Fig. 1B and C). The Von Frey test over the left affected hind limb revealed a significant decrease in withdrawal forces for both the control (CCI–UHF) and treatment (CCI ​+ ​UHF) groups at day 0 (n ​= ​6 per group; control vs. treatment; 0.45 ​± ​0.068 ​g vs. 0.48 ​± ​0.087 ​g, respectively), compared to the non-injured baseline at day −7 (control vs. treatment; 5.77 ​± ​1.39 ​g; 6.86 ​± ​1.40 ​g) (Fig. 1D). The withdrawal forces of the treatment group returned to near-baseline levels when measured 30 ​min following treatment (8.64 ​± ​0.73 ​g) and remained higher than day 0 levels up to day 3 following TENS treatment (10.25 ​± ​2.68 ​g) (Fig. 1D). On day 4, the rats in the treatment group again exhibited mechanical allodynia (0.55 ​± ​0.20 ​g). Therefore, the second TENS session was administered, and the withdrawal forces significantly and immediately returned to baseline (8.53 ​± ​0.89 ​g). The alleviation of mechanical allodynia was maintained for 4 days after the second TENS treatment, but mechanical allodynia reappeared on day 5 (0.34 ​± ​0.17 ​g) (Fig. 1D). In contrast, the withdrawal forces remained unchanged in the control group for 2 weeks (Fig. 1D).

### UHF-TENS maintains normal sensation without damage to myelinated axons in the uninjured nerve

To further confirm the potential effect on healthy nerves, UHF was also introduced to the uninjured sciatic nerve, which had only received nerve exposure surgery as a sham group. Two sessions of stimulation were administered to the uninjured sciatic nerve using the same protocol (Fig. 1C). This revealed that the withdrawal forces remained unchanged when compared to the uninjured nerve group without stimulation (Fig. 1E). We further investigated axon demyelination of the uninjured nerves at day 14 after the first session of stimulation, designated as the sham ​+ ​UHF group, while the uninjured nerves without electrical stimulation were designated as the sham-UHF group and were also extracted on the same day. Immunofluorescence staining (IF) of the left sciatic nerve revealed no significant difference in signals for MBP (Myelin Basic Protein) and NF-200 (Neurofilament-200). Additionally, the signal for MBP/NF200 colocalization remained identical in the TENS group as compared to the control group (Fig. 1F and G).

### UHF-TENS reduces pain neuropeptide and ameliorates neuropathic pain signals in the ipsilateral DRG

**Fig. 2:**
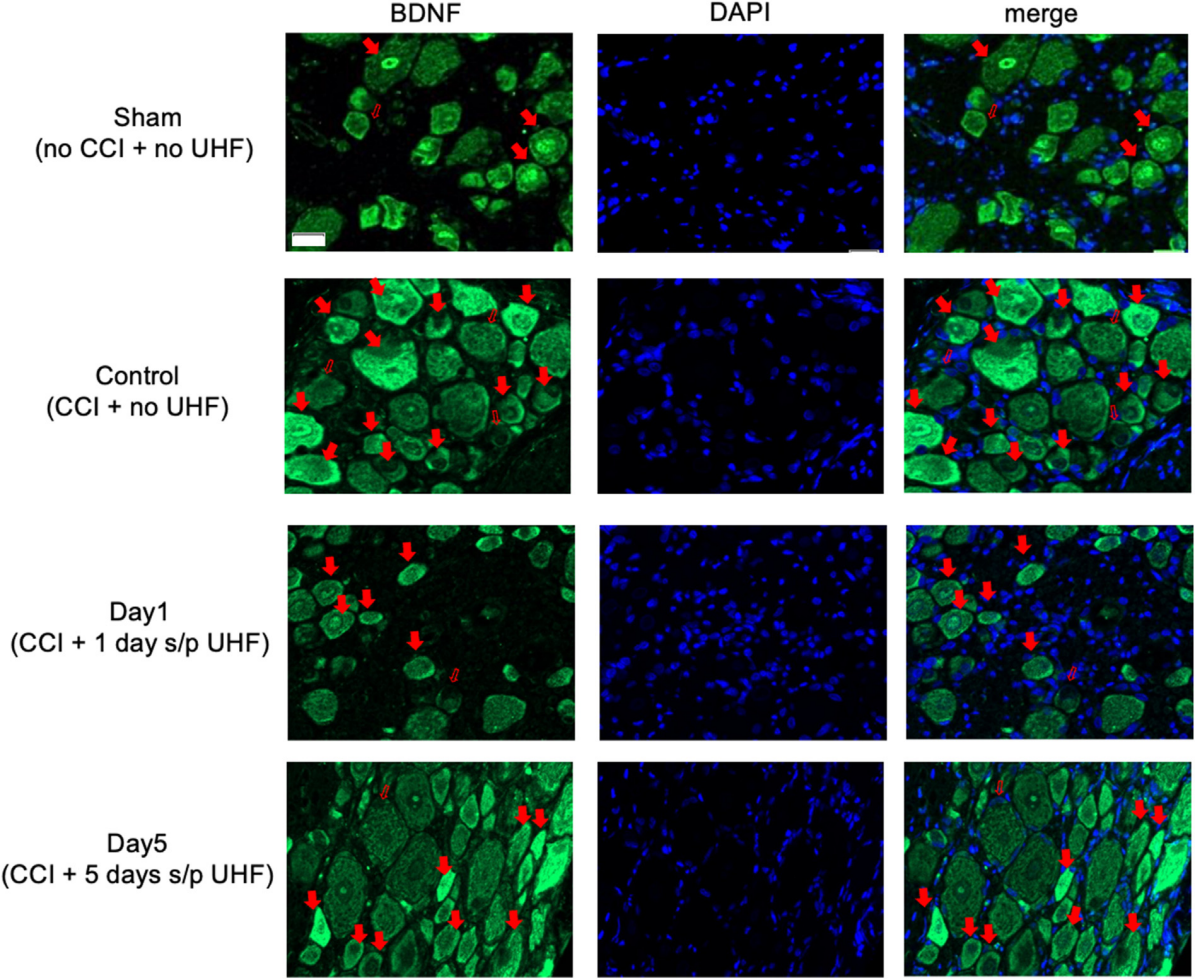
Immunofluorescent staining of BDNF in the injured side of dorsal root ganglion under UHF-TENS treatment. A significant increase in signal intensity was induced by nerve constriction injury in the control group compared to sham group as the baseline. After UHF stimulation, the signal intensity of BDNF decreased significantly on Day 1 and returned to baseline level on Day 5. (n ​= ​4 per group; Scale bar: 20 ​μm; positive neuron: red solid arrowhead; negative neuron: red hollow arrowhead).

**Fig. 3:**
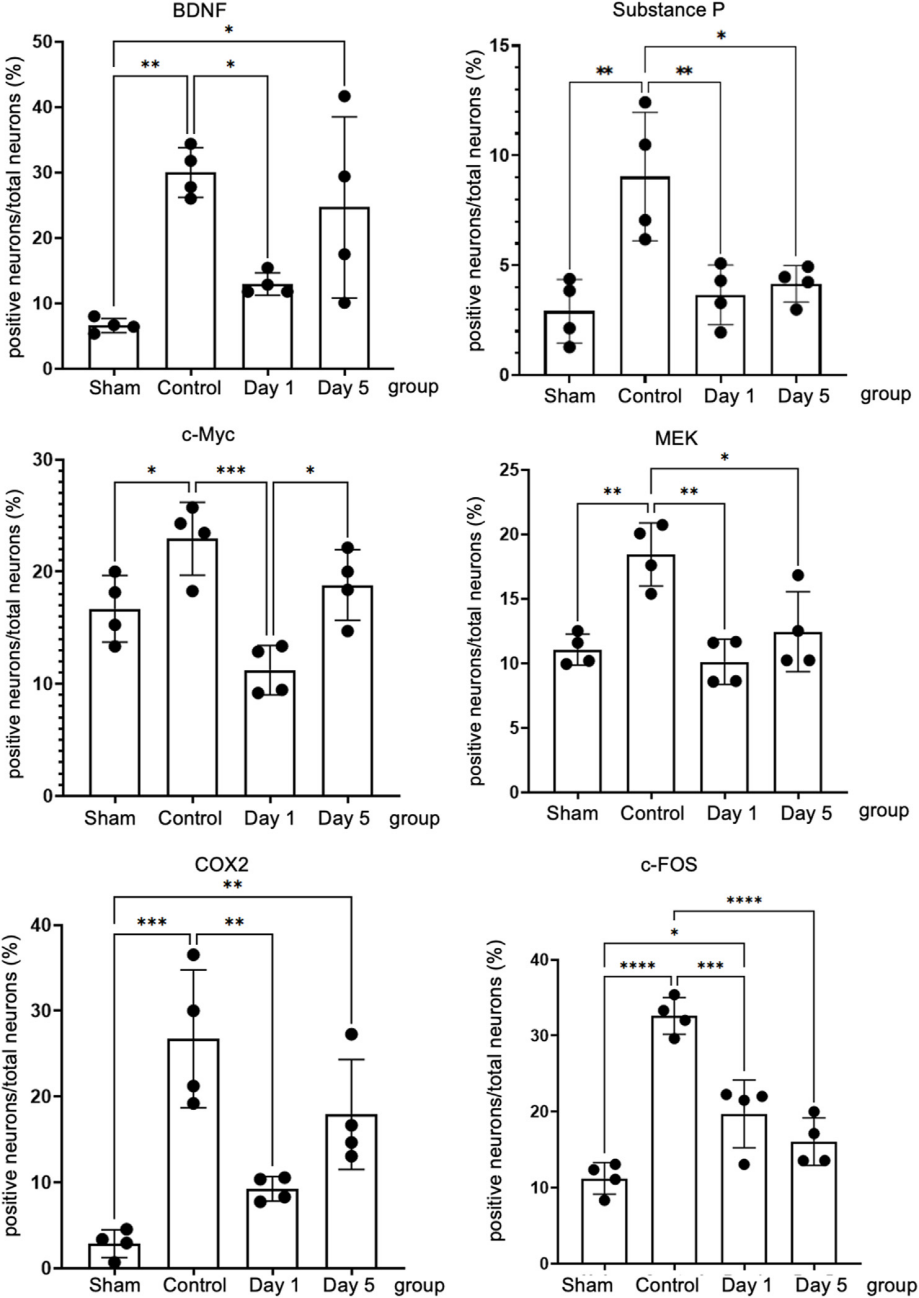
Quantitative analysis of immunofluorescent staining of neuropathic pain markers on the injured side of dorsal root ganglion. BDNF (brain-derived neurotrophic factor), cyclooxygenase 2 (COX2), and c-Myc significant increase in signal intensity were induced after nerve injury in the control group compared to the uninjured nerve as the baseline. After UHF stimulation on Day 1, the signal intensity decreased and returned to increase on day five after electrical stimulation therapy. Similarly, substance P (SP), MEK, and c-Fos over injured DRG significantly increased signal intensity after nerve injury in the control group compared to the uninjured nerve as the baseline. After electrical stimulation therapy on Day 1, the signal intensity decreased and still reduced compared to the injured nerve of the control group on day five after UHF stimulation. (n ​= ​4 per timepoint Data was presented with mean ​± ​standard deviation, ∗ indicated p ​< ​0.05; ∗∗ indicated p ​< ​0.01, ∗∗∗ indicated p ​< ​0.001, ∗∗∗∗ indicated p ​< ​0.00001, and ns indicated no significant difference).

**Figure S1:**
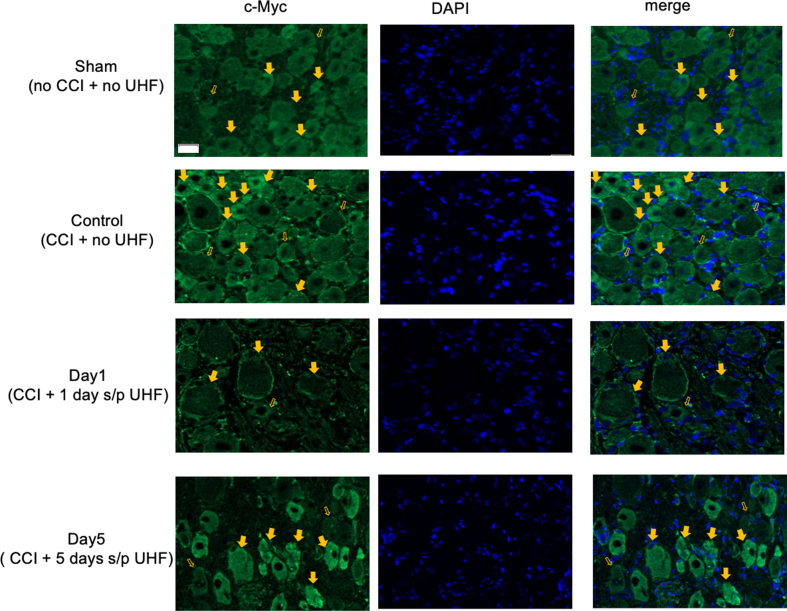
Immunohistochemical staining of c-Myc in the injured side of dorsal root ganglion under UHF-TENS treatment. A significant increase in signal intensity was induced by nerve constriction injury in the control group compared to sham group as the baseline. After UHF stimulation, the signal intensity of c-Myc decreased significantly on Day 1 and returned to baseline level on Day 5. (n=3-4 per group; 20 μm; positive neuron: yellow solid arrowhead; negative neuron: yellow hollow arrowhead).

**Figure S2:**
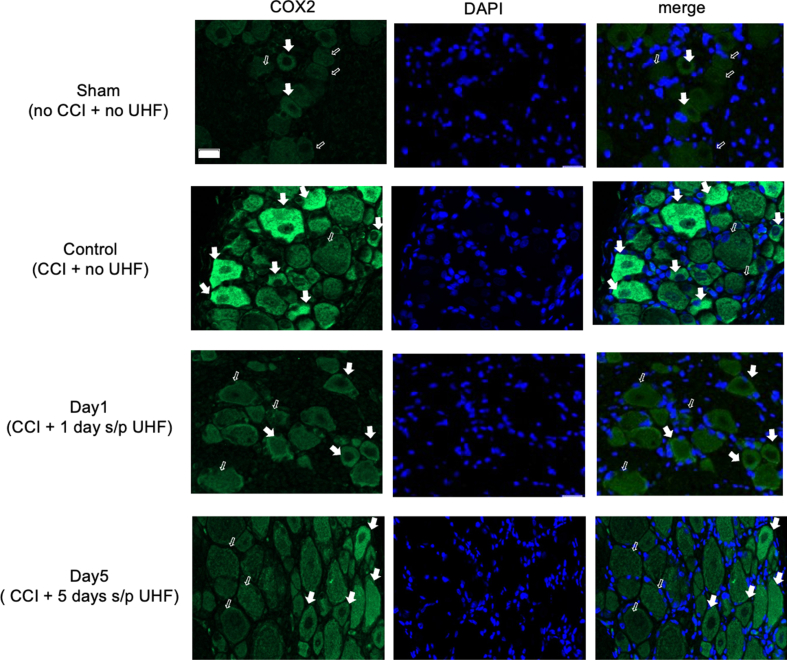
Immunohistochemical staining of COX2 in the injured side of dorsal root ganglion under UHF-TENS treatment. A significant increase in signal intensity was induced by nerve constriction injury in the control group compared to sham group as the baseline. After UHF stimulation, the signal intensity of COX2 decreased significantly on Day 1 and returned to baseline level on Day 5. (n=3-4 per group; Scale bar:20 μm; positive neuron: white solid arrowhead; negative neuron: white hollow arrowhead).

**Figure S3:**
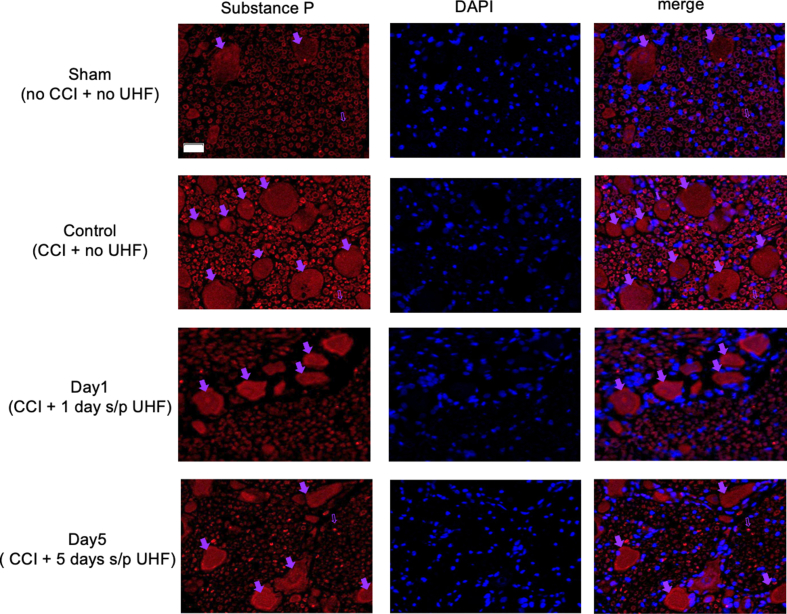
Immunohistochemical staining of Substance P in the injured side of dorsal root ganglion under UHF-TENS treatment. A significant increase in signal intensity was induced by nerve constriction injury in the control group compared to sham group as the baseline. After UHF stimulation, the signal intensity of Substance P decreased significantly on Day 1 through Day 5. (n=4 per group; Scale bar: 20 μm; positive neuron: purple solid arrowhead; negative neuron: purple hollow arrowhead).

**Figure S4:**
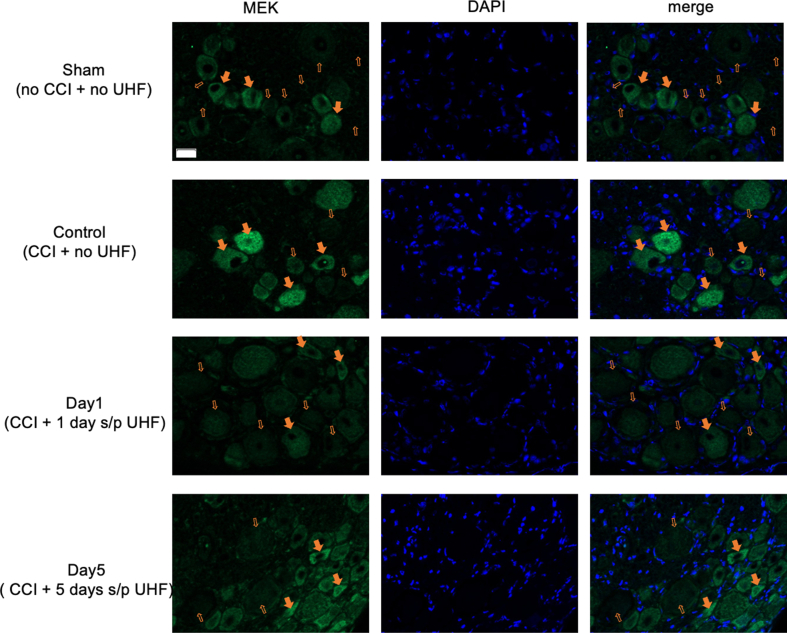
Immunohistochemical staining of MEK in the injured side of dorsal root ganglion under UHF-TENS treatment. A significant increase in signal intensity was induced by nerve constriction injury in the control group compared to sham group as the baseline. After UHF stimulation, the signal intensity of MEK decreased significantly on Day 1through Day 5. (n=4 per group; Scale bar: 20 μm; positive neuron: orange solid arrowhead; negative neuron: orange hollow arrowhead).

**Figure S5:**
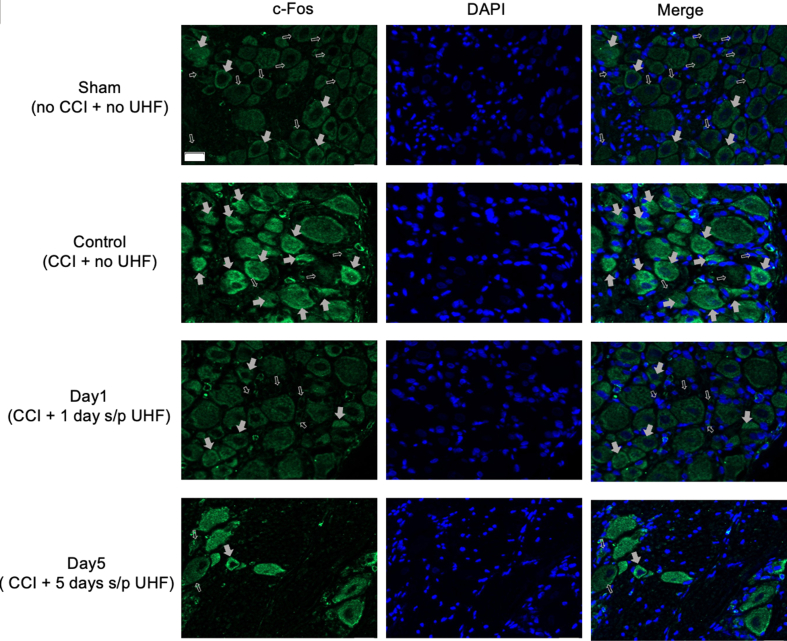
Immunohistochemical staining of c-Fos in the injured side of dorsal root ganglion under UHF-TENS treatment. A significant increase in signal intensity was induced by nerve constriction injury in the control group compared to sham group as the baseline. After UHF stimulation, the signal intensity of -Fos decreased significantly on Day 1 through Day 5. (n=4 per group; Scale bar: 20 μm; positive neuron: grey solid arrowhead; negative neuron: grey hollow arrowhead).

To further explore the direct effect of neuromodulation through UHF stimulation, pain-related neuropeptides and neuroinflammatory signals in the L3–L5 DRG were investigated on days 1 and 5 after treatment and compared with the control group without treatment. In the injured side of the DRG, pain-related signals including BDNF, COX2, and c-Myc were highly expressed in the control group after peripheral nerve injury, with percentages of 29.98% ​± ​3.79%, 26.75% ​± ​8.04%, and 22.94% ​± ​3.25%, respectively. However, in the treatment group, these signals significantly decreased after day 1 (12.95% ​± ​1.71%, 9.25% ​± ​1.44%, and 11.22% ​± ​2.20%, respectively). By day 5, BDNF signals had returned to baseline (24.66% ​± ​13.85%) compared to those in the injured nerve of the control group (Fig. 2, Fig. 3). Furthermore, the other two pain-related peptides, c-Myc and COX2, showed a similar trend to that of BDNF (18.81% ​± ​3.14% and 17.92% ​± ​6.41%, respectively) (Fig. 3; Supplementary Figs. 1 and 2). Additionally, other neuropathic pain signals, such as SP (Substance P), MEK, and c-Fos in the DRG on the injured side, also exhibited high expression in the control group (9.04% ​± ​2.92%, 18.44% ​± ​2.44%, and 32.60% ​± ​2.41%, respectively) after nerve injury compared to the sham group. After UHF stimulation, these signals significantly decreased on day 1 (3.66% ​± ​1.36%, 10.11% ​± ​1.75%, and 19.71% ​± ​4.45%, respectively) and continued to decrease until day 5 (4.16% ​± ​0.83%, 12.45% ​± ​3.11%, and 16.06% ​± ​3.12%, respectively) (Fig. 3; Supplementary Figs. 3, 4, and 5).

### UHF-TENS modulates gene expression in the ipsilateral DRG

**Fig. 4:**
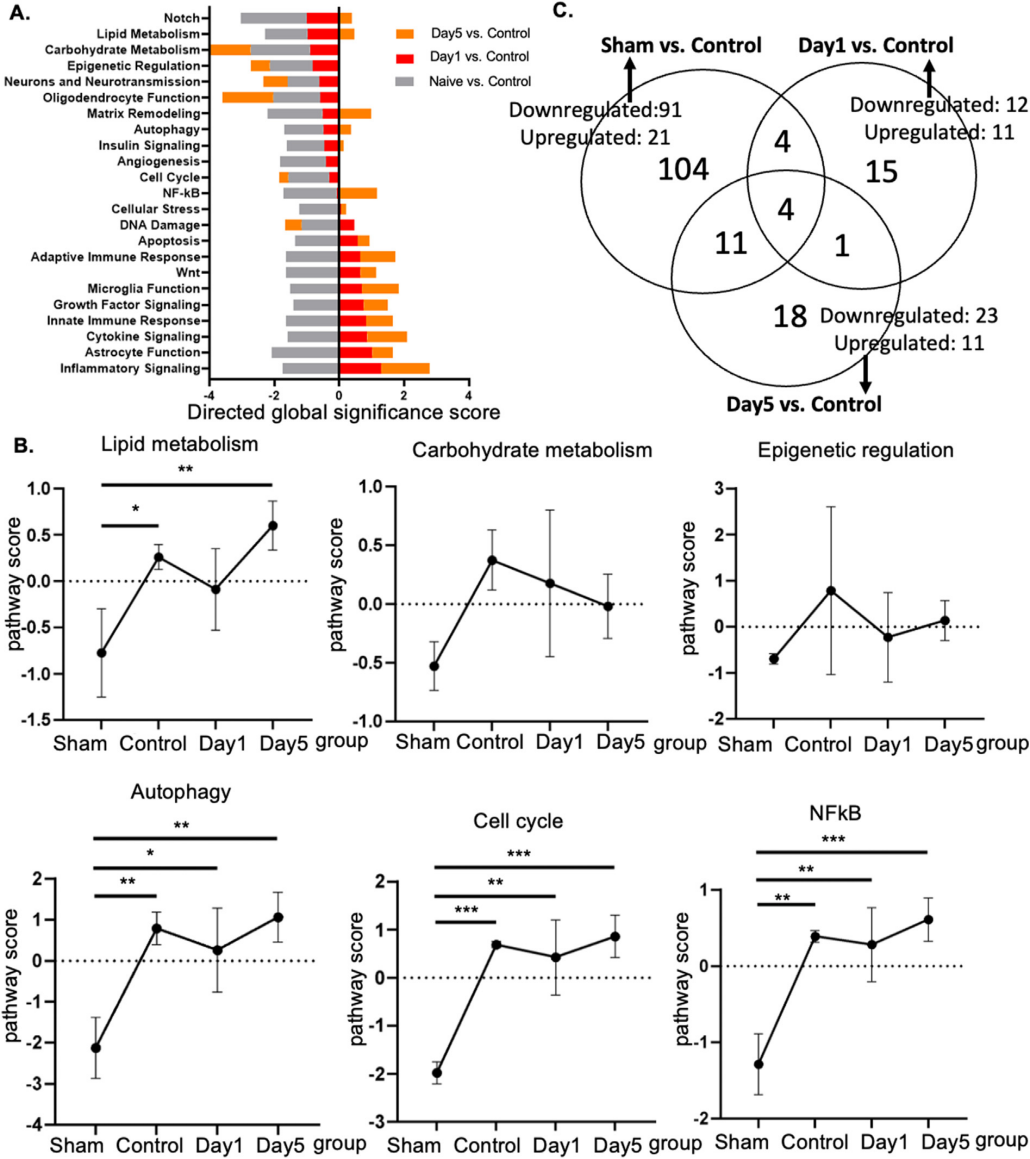
Genetic profiling of injured nerve modified by UHF stimulation by Nanostring nCounter Neuroinflammation panel (A) Neuroinflammation pathway-specific changes in gene differential expression among sham group, control group, and UHF treated nerve at Day 1 and Day 5 by directed global significance score. (B) The pathway scores of lipid metabolism, carbohydrate metabolism, epigenetic regulation, autophagy, cell cycle, and NFkB were upregulated after peripheral nerve injury and downregulated after UHF treatment. (C) Venn diagram showing the overlap of the significant differential expression gene lists for the three pairwise comparison. The overlap of Sham vs. Control and Day1 vs. Control area identifies eight genes that are both modified by injury as well as UHF treatment after CCI. (n ​= ​3 for each group).

To further investigate the underlying transcriptional regulation in the DRG by UHF electrical stimulation, the L4–L5 DRGs from the injured site of the nerve were harvested, and the RNA expression levels were further analyzed using NanoString's nCounter technology on day −7 (no nerve injury as the sham group) and day 0 (nerve injury as control), including days 1 and 5 after UHF stimulation. Based on the directed global significance score among 23 neuroinflammation pathways, altered gene expressions in the DRG that were downregulated after treatment were strongly related to lipid metabolism, carbohydrate metabolism, epigenetic regulation, autophagy, cell cycle, and NF-κB (Fig. 4A and B).

**Fig. 5:**
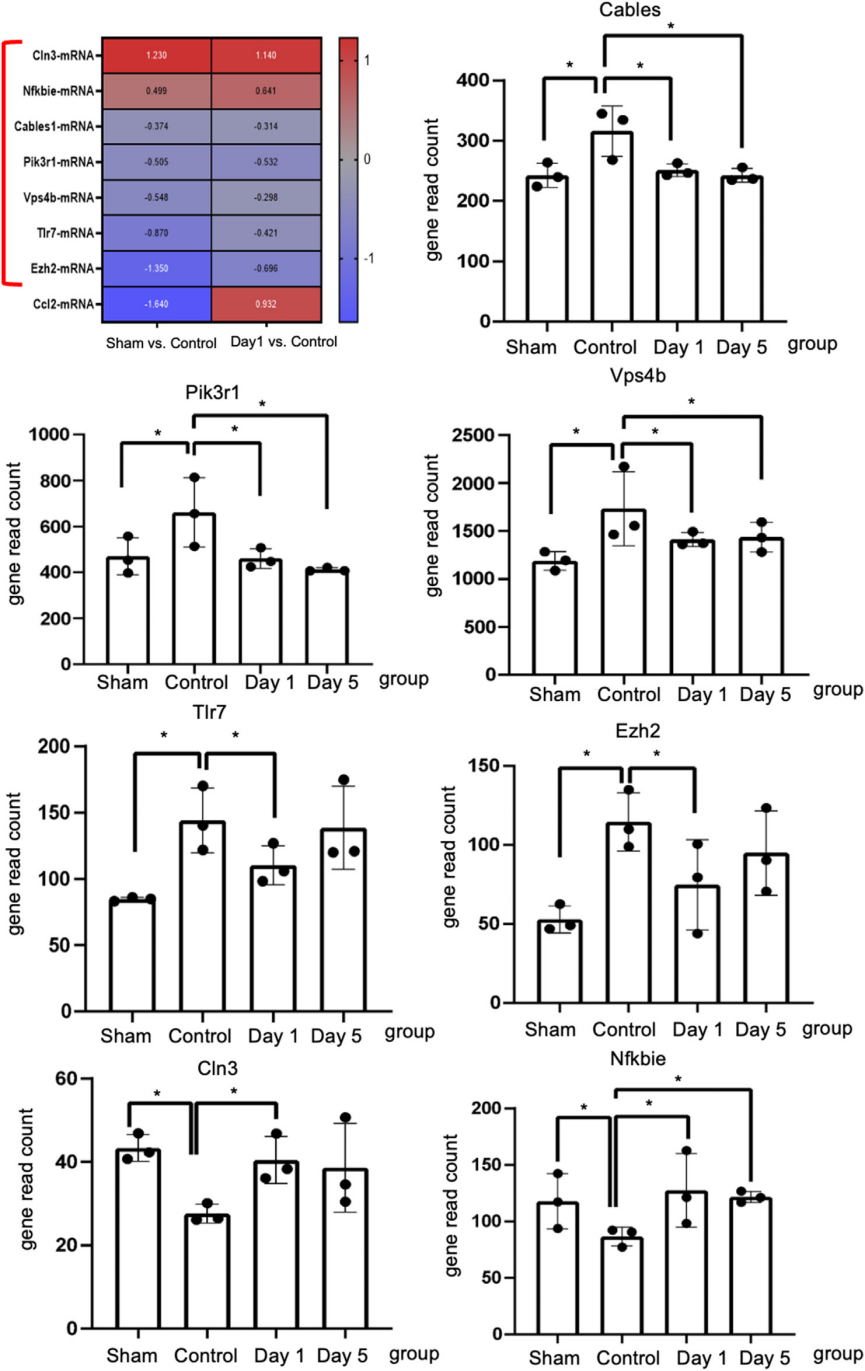
The Nanostring gene analysis of inflammatory genes in the dorsal root ganglion. The heatmap shows eight significant differential expression gene lists overlap genes between comparisons Day −7 (sham) vs. Day 0 (control) and Day 1 (UHF) vs. Day 0 (control). Among these, the gene expression of the below 7 genes, Cln3, Nfkbie, Cables1, Pik3r1, Vps4b, Tlr7, and Ezh2, indicate the potential UHF regulatory effects. The Nfkbie and Cln3 were upregulated after nerve injury in control group, but significantly downregulated by UHF treatment. The Tlr7, Pik3r1, Eh2, Vps4b, and Cables were downregulated after nerve injury in control group, while upregulated by UHF treatment. (n ​= ​3 for each group; ∗ indicated p ​< ​0.05).

**Figure S6:**
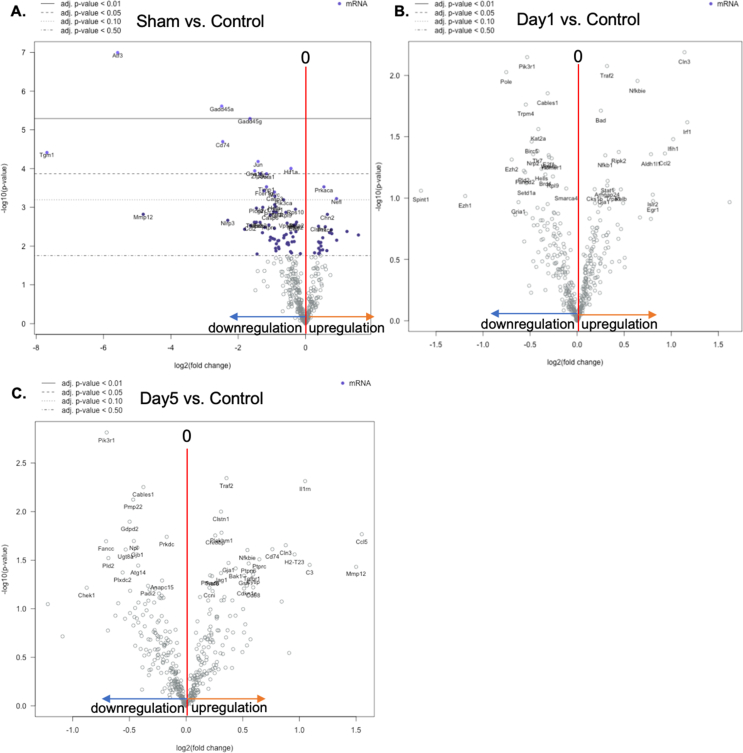
(A–C) Volcano plot of differential gene expression at sham group, Day 1, and Day 5, comparing with control group. (n=4 for each group).

Further investigation revealed a robust number of differentially expressed (DE) genes between groups (p-value <0.05). To identify the regulatory genes affected by UHF stimulation after nerve injury, two comparisons were analyzed: (1) sham vs. control: comparison 1; (2) day 1 vs. control: comparison 2; (3) day 3 vs. control: comparison 3. In comparison 1, CCI contributed to a significant differential expression of 112 genes (Fig. 4C). Among them, 91 genes were downregulated, and 21 genes were upregulated. In comparison 2, UHF led to a significant differential expression of 23 genes, with 12 genes being downregulated and 11 genes being upregulated. In comparison 3, UHF also led to a significant differential expression of 34 genes, with 23 genes being downregulated and 11 genes being upregulated. Then, the overlap of the DE gene lists between sham vs. control and day 1 vs. control was compared to determine which genes were modified by UHF stimulation, identifying a total of eight common genes in both comparisons. Among them, four genes were persistently significantly regulated until day 5 (Fig. 4C). Additionally, out of the eight common genes, five genes were upregulated in the control group compared to both the sham and UHF-day 1 groups, such as Cables, Pik3r1, Vps4b, Tlr7, and Ezh2 (Fig. 5). Conversely, two genes were significantly downregulated in the control group in both comparisons, such as Cln3 and Nfkbie (Fig. 5). The normalized gene read count of these seven genes demonstrated that UHF stimulation significantly downregulated Cables, Pik3r1, Vps4b, Tlr7, and Ezh2, while the expression of Cln3 and Nfkbie was upregulated on day 1 (Fig. 5). The similar distribution of significant differential gene expression could be identified by the volcano plot (Supplementary Fig 6).

## Discussion

**Fig. 6:**
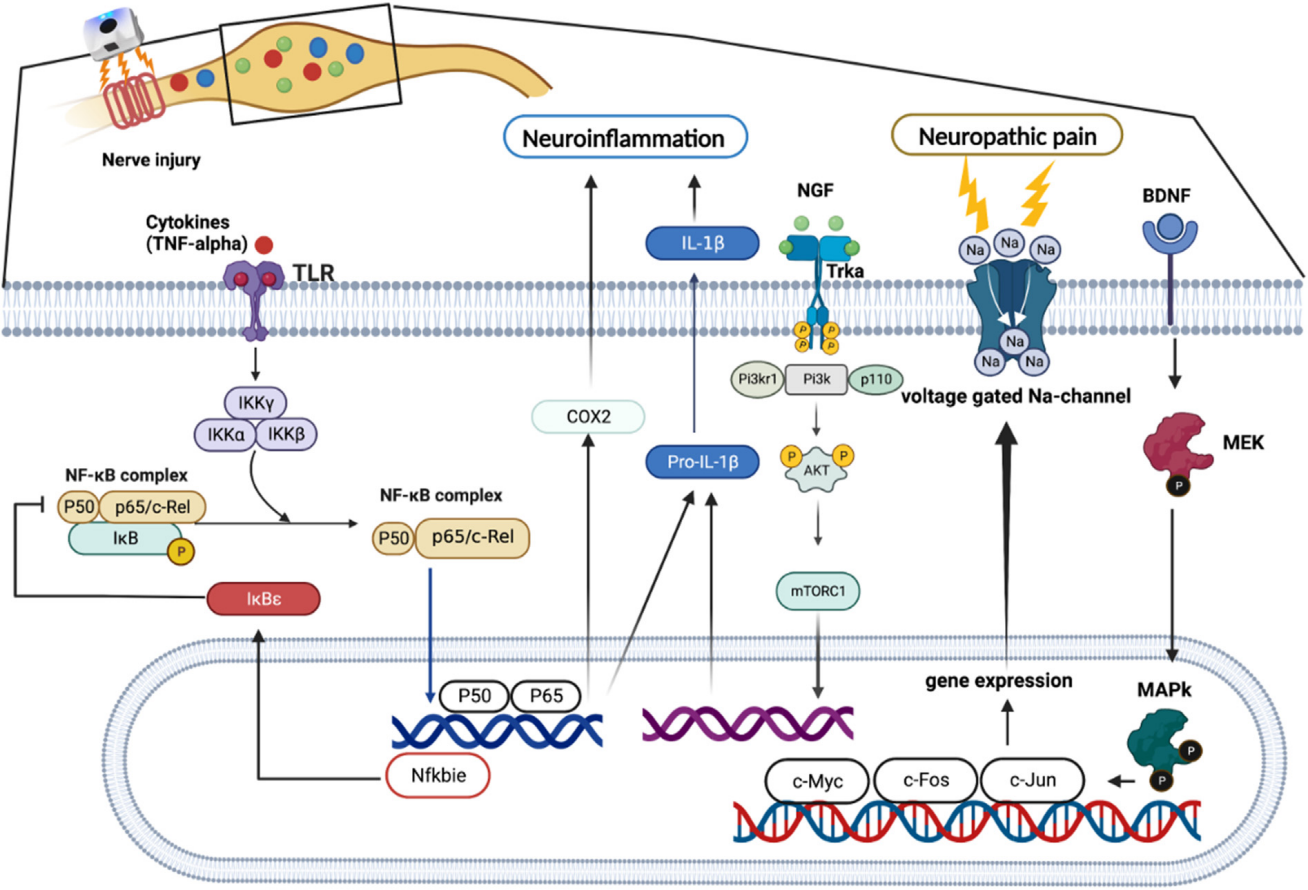
Sketch map of the regulatory molecular mechanism of UHF-TENS for neuropathic pain in DRG neuron of CCI rats, in terms of neuropathic pain formation and neuroinflammation signal modulation.

In this study, we demonstrated that UHF-TENS alleviates neuropathic pain in an animal model of peripheral compressive neuropathy, and our study also showed no noticeable nerve damage in the treated peripheral nerve (Fig. 1F and G). Furthermore, possible mechanisms are mentioned below and summarized in Fig. 6. We found that a significant decrease in pain-related neuropeptides and inflammatory markers, including MEK, c-Myc, c-FOS, COX2, and substance P, was observed in the damaged DRG neurons after electrical stimulation. Additionally, neuroinflammatory gene regulation was involved in the Tlr7-, Nfkbie-, and Pik3r1-mediated neuropathic pain pathway [12]. The aforementioned therapeutic effects and the understanding of the possible underlying regulatory mechanisms demonstrate that UHF-TENS is a promising modality for alleviating neuropathic pain.

It is well-known that BDNF is critical for neuronal survival, differentiation, and modulation of synaptic strength. It is categorized as a neuromodulator and plays a significant role in spinal plasticity. It is evident that BDNF's actions are diverse, as it can facilitate both adaptive and maladaptive plasticity within spinal networks [29]. The local and systemic growth factors BDNF is produced during an inflammatory reaction induced by nerve injury, and BDNF ligand receptor internalization mediates activation and phosphorylation of MAPK, creating a kinase complex (consisting of G-proteins Ras, Raf and MEK). Then, component of this activated MAPK (c-MAPK) translocates to the nucleus resulting in elevated expression of transcription factors c-Myc, Elk-1, c-FOS and c-Jun, and caused hyperesthesia eventually [12,30]. Nonetheless, certain studies have discovered that diminished BDNF levels, rather than increases, are linked to functional impairments and heightened mechanical sensitivity following spinal cord injury (SCI). Conversely, elevated BDNF levels following SCI contribute to adaptive plasticity and enhanced functional recovery. The diverse impacts of BDNF after SCI might be attributed to alterations induced by SCI in: (i) the expression of TrkB receptor and downstream kinases, (ii) GABA transmission resulting from changes in chloride transporter expression, and (iii) the activity of astroglial cells [31].

In this study, we found that the expression of BDNF and its downstream signaling proteins, such as MEK, c-Myc, and c-FOS, were downregulated in DRG after UHF-TENS treatment in a CCI animal model. This might suggest that UHF-TENS has the potential to relieve mechanical allodynia by influencing the pain transduction pathway through BDNF/MAPK modulation. (Fig. 2, Fig. 3). In a previous study, researchers reported that neuropathic pain may be relieved by other kinds of electrical stimulation, such as electroacupuncture, via the BDNF/MAPK signaling pathway regulation [32,33]. For the expression of COX2, it is implicated in the development and maintenance of neuropathic pain [34,35]. COX2 inhibition in infiltrating macrophages at the site of injury leads to changes in gene expression in the dorsal root ganglia, providing pain relief [36]. Furthermore, the production of prostaglandins, which are synthesized by COX enzymes, is involved in the pathogenesis of neuropathic pain. Thus, the downregulation COX2 expression might play a critical role in alleviating neuropathic pain. In addition, the expressions of substance P, which can bind to its specific receptor, neurokinin-1 (NK-1), to sensitize neurons and produce pain sensation [37,38], MEK, and c-FOS, which are the downstream protein of BDNF/MAPK pathway [12], were persistently downregulated until 5 days after electrical treatment. This might suggest that the electrical stimulation might alleviate neuropathic pain by prolonging its inhibition on the expression of substance P, MEK, and c-FOS.

To further explore the differential gene expression profile in neuroinflammation, we found that the mRNA expression levels of Cln3 and Nfkbie were both upregulated from days 0–1 post-treatment (Fig. 5). For Nfkbie, the protein encoded by this gene binds to p50 of the NF-κB complex, trapping the complex in the cytoplasm and preventing it from activating genes in the nucleus. The related NF-κB p65 was upregulated in CCI rats, indicating that it might exacerbate neuropathic pain by inducing an inflammatory response [39] Thus, we could speculate that the increased expression of Nfkbie might alleviate neuropathic pain by inhibiting the activation of NF-κB; however, further validation is warranted. The Cln3 gene encodes a protein that is involved in lysosomal function and causes a neurodegenerative disease commonly known as Batten disease. The inflammasome is a key molecular pathway for activating pro-IL-1β in microglia, and elevated IL-1β can induce neuronal cell death. A previous study elucidated that microglia deficient in the Cln3 gene showed increased expression of inflammasome activation and IL-1β release compared to wild-type microglia under neuronal damage conditions. This suggests that Cln3-deficient neurons are less equipped to withstand cytotoxic insults generated by activated microglia [40].

In contrast, the reverse tendency was observed in several signaling genes, where downregulation was observed in Tlr7, Pik3r1, Ezh2, Vps4b, and Cables after UHF-TENS treatment. For Tlr7, the protein encoded by this gene is a member of the Toll-like receptor family, which plays a fundamental role in pathogen recognition and activation of innate immunity. In a previous study, CCI of sciatic nerve induced a robust increase of TLR7 at mRNA and protein levels in mouse ipsilateral DRG [41]. It might suggest that TLR7 expression level within the injured DRG may contribute to neuropathic pain through the upregulation of the NF-κB pathway in primary sensory neurons. Hence, TLR7 may be a potential therapeutic target for neuropathic pain treatment. As for Pik3r1 gene, it encodes 85-kD regulatory subunit of phosphoinositide 3-kinase (PI3K), which is important for many cell activities, including cell growth and division, cell migration, production of new proteins, and cell survival. As for the role of PI3K signaling in neuropathic pain, a previous study revealed that the CCI procedure could induce PI3K/Akt signaling in the DRG and spinal cord, and the blockage of PI3K signaling could alleviate CCI-evoked allodynia [42]. Thus, PI3K signaling might be required for spinal central sensitization in the CCI neuropathic pain model. The EZH2 gene, which is a histone methyltransferase, catalyzes the methylation of histone H3 on K27 (H3K27), causing gene silencing. As for its effect in neuropathic pain, previous research demonstrated that nerve injury led to an increased number of neurons with EZH2 expression. More strikingly, nerve injury remarkably increased the number of microglia with EZH2 expression [43]. Other studies elucidated that EZH2 was the downstream gene of miR-378 and was negatively regulated by miR-378. The upregulation of EZH2 expression in the CCI rats may further ameliorate the inhibitory effects of miR-378 during neuropathic pain evolution [44]. Thus, the miR-378/EZH2 axis may be a novel target in terms of theranostics for clinical neuropathic pain patients. However, current studies only identified its function in microglia; therefore, the role of EZH2 in DRG still warrants further exploration. The Vps4b gene encodes the protein, which is a member of the AAA protein family (ATPase associated with diverse cellular activities). This associates with endosomal compartments and is involved in intracellular protein trafficking. In a previous study, Vps4b expression was highly increased in the hippocampus CA1 subregion after middle cerebral artery occlusion, reaching its peak after 3 days, and the expression of active caspase-3 was promoted, which could induce apoptosis [45]. Thus, it might play an important role in promoting neuronal apoptosis. Cables1 gene encodes the binding protein that can link Cdk5 and c-Abl and also plays a role in the proliferation, cell differentiation, and regulation of axon growth. Furthermore, active c-Abl kinase leads to Cdk5 tyrosine phosphorylation, and this phosphorylation is enhanced by Cables [46]. In the peripheral nerve system, Cdk5 might contribute to the development and maintenance of neuropathic pain, either enhancing protein trafficking to the plasma membrane or by aiding in the development of morphine nociceptive tolerance, as evidenced in a previous review article [47].

Some limitations of this study should be noted. Firstly, we administered anesthesia only to the treatment group during electrical stimulation, while the control and sham groups were not given anesthesia. Thus, we did not consider the potential impact of isoflurane on mechanical allodynia. Secondly, we did not employ further quantitative assays, such as Western blotting, to validate our findings because the sample quantity of DRG was insufficient to provide staining for such a multitude of markers.

UHF-TENS contributes to the alleviation of mechanical allodynia in an animal model of chronic constriction injuries, with no additional axon damage. The potential molecular mechanism of UHF-TENS results from the regulation of pain-related neuropeptides, such as BDNF, COX2, c-Myc, SP, c-Fos, and MEK, along with the regulation of neuroinflammatory genes, such as Cables, Pik3r1, Vps4b, Tlr7, Ezh2, Cln3, and Nfkbie in injured DRG neurons. Future proteomic investigations should be performed to validate specific pain-related pathways with quantitative assessments, such as IL-1β. Additionally, we should further optimize the repetitive treatment protocols of UHF-TENS to accommodate various clinical conditions. Furthermore, in combination with the regulation of neuroinflammatory genes, UHF-TENS could become a new modality to enhance the treatment of neuropathic pain in the future.

## Authors’ contributions

SC and YL are the authors who performed experiments and collect data; CW, SL and YH developed of the concepts, designed the experiments; WT and WL provided system and technical supports, discussion and problem solving in researches; SC and YL wrote the manuscript; YH revised the manuscript and organized the current study. All authors have read and agreed to the published version of the manuscript.

## Ethics approval

Animal protocols and surgical procedures such as animal housing and care were approved by the Laboratory Animal Center and Institutional Animal Care and Use Committee (IACUC, Approval number 111224) at National Cheng Kung University (Tainan, Taiwan).

## Availability of data and materials

The datasets analyzed for this study are publicly available in article. Further inquiries can be directed to the corresponding author.

## Funding

This study was supported in part by grants from the National Science and Technology Council (111-2311-B-006 -001 and 111-2923-B-006-001-MY2 to YH; 111-2314-B-006 -095 to SC) in Taiwan and Gimer Medical Co., Ltd. (New Taipei city, Taiwan). The funders were not involved in the study design, collection, analysis, interpretation of data, the writing of this article or the decision to submit it for publication. All authors declare no other competing interests in Taiwan.

## Declaration of competing interest

The authors declare the following financial interests/personal relationships which may be considered as potential competing interests: Yuan-Yu Hsueh reports financial support was provided by National Science and Technology Council. Szu-Han Chen reports financial support was provided by National Science and Technology Council. Wei-Tso Lin reports a relationship with Gimer Medical Co., Ltd, New Taipei City, Taiwan that includes: employment. If there are other authors, they declare that they have no known competing financial interests or personal relationships that could have appeared to influence the work reported in this paper.

## Appendix A Supplementary data

Multimedia component 1 The number of animals in each group of this study. Multimedia component 1
