# Supplementary material for: Ultrahigh frequency transcutaneous electrical nerve stimulation for neuropathic pain alleviation and neuromodulation
Source: Neurotherapeutics. 2024 Feb 16;21(3):e00336. doi: 10.1016/j.neurot.2024.e00336 (PMC10943071; doi:10.1016/j.neurot.2024.e00336)
Supplement: Multimedia component 1 — The number of animals in each group of this study. [file mmc1.docx]

**Supplementary Table 1.** The number of animals in each group of this study

|  | **two-session therapy** | **one-session therapy** | |
| --- | --- | --- | --- |
|  | von Frey test | IF (nerve^a^) | IF/Nanostring (DRG^b^) |
| **Biosafety test** | | | |
| Sham-UHF | 3 (figure 1E) | 4^c^ (figure 1G) | - |
| Sham+UHF | 3 (figure 1E) | 3^c^ (figure 1G) | - |
| Total number (rat) | 6 | 7 | - |
| **Definite treatment** | | | |
| CCI-UHF | 6 (figure 1D) | - | - |
| CCI+UHF | 6 (figure 1D) | - | - |
| Sham | - | - | 4/4 (figure 3&5) |
| Control | - | - | 4/4 (figure 3&5) |
| Day 1 | - | - | 4/4 (figure 3&5) |
| Day 5 | - | - | 4/4 (figure 3&5) |
| Total number (rat) | 12 | - | 32 |

a. The rats were sacrificed for sciatic nerve collection

b. The rats were sacrificed for DRG collection

c. The rats in both group were sacrificed on 14 days after UHF therapy

IF:Immunofluorescent staining; DRG:dorsal root ganglion; UHF:ultrahigh frequency
